# Intelligent identification of rice leaf diseases via improved faster-RCNN with multi-feature scale fusion

**DOI:** 10.1371/journal.pone.0345005

**Published:** 2026-03-26

**Authors:** Xiaofan Shi, Wei Zhang, Fang Song, Chunfeng Zhao

**Affiliations:** The Engineering Training Center, Shanghai University of Engineering Science, Shanghai, China; Universidade Federal de Uberlandia, BRAZIL

## Abstract

Many Artificial Intelligence and Machine Learning technologies have been applied to detect rice diseases. These approaches are either unable to identify the diseases or have a slow recognition speed. Therefore, an improved Faster-RCNN (Faster-RCNN-Pro) model is proposed to overcome these issues. First, SENet attention modules are embedded in the backbone of Faster-RCNN to enhance confidence of objects that are difficult to recognize by enhancing key image information and suppressing background information. Second, structure of the feature extraction network and RPN are improved by using multi-feature scale fusion to increase the utilization of micro-target features. Third, the quantization error introduced in the process of pooling the region of interest is then eliminated by ROI Align. Finally, a balanced L1 loss function is designed to effectively reduce the imbalance between samples with a large gradient that are difficult to learn, and samples with a small gradient that are easy to learn. The experiment results show that the improved model has a better detection accuracy and robustness in recognizing the fine features of rice leaf diseases. Therefore, the application of this model to the intelligent identification of rice leaf disease can significantly improve the accuracy and reduce the misjudgment rate.

## Introduction

Rice serves as a primary food source across the globe and underpins the livelihoods as well as the economic development of millions of people. In numerous developing countries, rice not only supplies the food and energy required by people but also plays a constructive role in facilitating employment and driving economic growth in rural areas. However, disease-related problems frequently destroy a considerable rice volume due to a lack of adequate field monitoring.

Approximately 37% of rice crops are lost to pests and diseases annually by farmers [1]. There exist several approaches to mitigate the issue of rice loss due to diseases. These include thorough cleaning of equipment and fields between seasons, the employment of clean seeds and resistant varieties, the application of appropriate fertilizers, and the fostering of natural pest predators. Among these strategies, which are predominantly preventive in nature, there are instances where the use of insecticides becomes crucial, especially in combating rice leaf diseases. However, the variety of rice leaf diseases is extensive, and each demands a specific type of insecticide. Therefore, the selection of insecticides must be carefully tailored to the particular disease in question. Treatment should typically commence with insecticides that target the most prevalent rice leaf diseases in paddy fields. Subsequently, insecticides may be periodically rotated to address other diseases, a process whose duration can vary depending on the number and severity of the diseases present. It is expected that advancements in precise agricultural monitoring of rice leaf diseases will lead to more timely and accurate treatment with the appropriate insecticides, thereby enhancing the overall effectiveness of disease management and potentially reducing crop losses.

Generally, rice leaf diseases typically cover sheath blight, bacterial blight, and rice blast. Their symptoms are manifested in aspects like texture, color, and shape, and they are characterized by rapid occurrence and being prone to infection [2]. At present, farmers and experts often conduct visual inspections for rice diseases. Once rice diseases are spotted in paddy fields, farmers will promptly choose appropriate insecticides to safeguard the yield of rice growth. In the case where multiple rice diseases exist in a paddy field, farmers might select just a single type of insecticide that is only suitable for one or a few kinds of rice diseases to treat the whole paddy field. Although the majority of rice plants in the paddy fields are treated with insecticides, some may still get damaged, which will lead to a reduction in the rice growth yield. Consequently, it is of vital importance to accurately classify the types of rice diseases on each leaf so as to formulate an appropriate plan to protect rice plants from all diseases. If rice diseases can be precisely detected, zoning of paddy fields for appropriate insecticide treatment can be achieved to deal with all types of rice diseases. Hence, there is an urgent need to develop efficient and accurate methods for detecting rice diseases.

Aiming to enhance the accuracy as well as the speed of diagnosis, many researchers have dedicated themselves to the automatic diagnosis of rice diseases. Some of the approaches they have explored include computer vision [3], support vector machines (SVM) [4,5], digital image processing techniques investigated [6,7], and pattern recognition techniques [8]. It’s worth noting that all of these aforementioned methods fall into the category of conventional ones.

In the realm of agricultural research focusing on rice health assessment, a Support Vector Machine (SVM)-based model [9] has been proposed with the aim of classifying three distinct classes of rice diseases, namely blight, brown spot, and smut. The image data utilized for this study were captured directly from a rice farm. Through meticulous experimentation, this model demonstrated an accuracy rate of 93.33% when applied to the training dataset, while achieving 73.33% accuracy on the test dataset. Regarding the quantification of rice crop damage resulting from hopper infestation [10], the Fuzzy C means classifier was strategically implemented. This classifier was tasked with categorizing the infestation into four severity classes, specifically severe, moderate, mild, and no infestation. The outcomes of this approach revealed an accuracy level reaching 87%. In another research endeavor [11], an innovative technique was investigated to classify and detect various types of mineral deficiencies prevalent in rice crops. The model construction incorporated two different types of inputs, namely textual information, and color features, with varying numbers of neurons configured within the hidden layers. Subsequently, an accuracy of 88.56% was successfully attained. Furthermore, an alternative approach was put forward for the identification of blast and brown spot diseases on rice. The Fractal Fourier method [12] was exploited to formulate a methodology that analyzes the texture characteristics of the rice samples to facilitate disease identification. Specifically, the raw images were converted into the CIELab color space. Through this process, the developed system achieved an accuracy rate of 92.5%. The Gray-Level Co-occurrence Matrix (GLCM) technique also played a significant role in the classification of rice health status, determining whether the rice was healthy or infected by diseases. Numerous researchers in this field have been dedicated to improving both the accuracy and the speed of identifying rice diseases. To achieve this, they have resorted to traditional yet effective methods, including pattern recognition techniques, support vector machines, digital image processing techniques, and computer vision. In a particular study [13], the classification of images depicting infected rice was carried out using the Self Organizing Map (SOM). During the model training phase, the training images were generated by extracting the characteristic features from the infected portions of the rice leaves. Meanwhile, four other types of images were employed for testing purposes. The researchers harnessed the power of a Neural network to simulate and analyze the results. Notably, the utilization of the classifier led to an enhancement in the classification accuracy. Finally, a novel stacked Convolutional Neural Network (CNN) architecture was proposed [14]. This architecture incorporated a two-stage training mechanism, which not only significantly reduced the model size but also managed to maintain a commendable level of classification accuracy. Specifically, when this stacked CNN was employed in lieu of the VGG16 model, the test accuracy was determined to be an impressive 95%.

It’s worth noting that researchers have begun to shift away from these traditional methods and turn towards deep learning models for the detection of diseases in various plants [15–17]. Among the most effective image classification methods that have achieved significant success is the convolutional neural network (CNN), which is a deep learning technique [18–22]. Lu et al. [20] put forward a technique for identifying rice diseases by utilizing a deep CNN. Through a 10-fold cross-validation scheme, ten common rice diseases were identified, with an average recognition rate reaching 95.48%. The Faster R-CNN methodology proposed by Zhou et al. [23] appears to be the optimal approach for identifying rice diseases due to its high speed and accuracy. Another method proposed by Ren et al. [24] employed Faster R-CNN to enhance accuracy and detect plant diseases. In order to be suitable for monitoring large-scale plantations, it was necessary to reduce the time required for disease identification. Bari et al. [25] introduced an upgraded Region Proposal Network (RPN) architecture that can precisely locate objects to generate candidate areas, thus enabling real-time detection of rice leaf diseases through the improved Faster R-CNN. Training the Faster R-CNN model on publicly available online and real-field rice leaf datasets enhanced its robustness. Yuan et al. [26] have proposed a lightweight identification algorithm for weedy rice that is based on the improved YOLOV5.

The development of a technique for the automatic identification of rice leaf disease has hitherto encountered numerous challenges. It should be noted that both the diagnosis and detection entail processes that can make it extremely difficult to accurately segment the specific area where the symptoms appear within the rice plant. The capture conditions are tough to manage, which in turn can complicate the prediction of images and further impede the detection of the disease. Furthermore, the symptoms caused by different diseases may look identical visually, and the discrimination methods often rely on very subtle variations. Another common issue lies in the discrepancies regarding the distribution of data features for training the model and the data used for validating it. This situation gives rise to the overfitting problem, which is of great significance when plant diseases are automatically detected, as the symptoms can vary depending on geographical locations and thus fall into the overfitting trap. It has also been noticed that many of the proposed rice leaf disease diagnostic architectures are designed for off-line use, and only a few experiments have been conducted in real-time. Usually, enhancing the image resolution in real-time also leads to an increase in computational complexity. Additionally, the difficulty of real-time operations grows with a wide variety of disease features, complex backgrounds, and indistinct boundaries of the disease symptoms. To tackle these challenges, the current study attempts to utilize the deep learning approach based on an improved Faster-RCNN to carry out real-time detection of rice leaf diseases. This investigation aims to alleviate the persistent problems in the process of developing a system for diagnosing rice diseases. The key contributions of the research are summarized as follows:

(1) Prediction confidence in objects that are difficult to recognize is enhanced by embedding a SENet attention module into the backbone of Faster-RCNN.
(2) By multi-feature scale fusion, feature information of small objects can be mapped into the feature maps, and the feature maps contain richer semantic information, thereby improving detection accuracy.
(3) The problem of region mismatch is solved by using ROI Align instead of ROI pooling.
(4) A new loss function is developed to address the imbalance between difficult-to-learn samples with large gradients and easy-to-learn samples with small gradients.

The rest of this paper is structured in the following way. Section 2 presents a description of the dataset and the computational environment employed in the study. A detailed account of the proposed method is provided in Section 3. In Section 4, an evaluation of the method is conducted, and the factors that have an impact on its performance are analyzed. Finally, in Section 5, we draw conclusions for our paper and put forward potential topics for future research.

## 1 Materials

**Fig 1:**
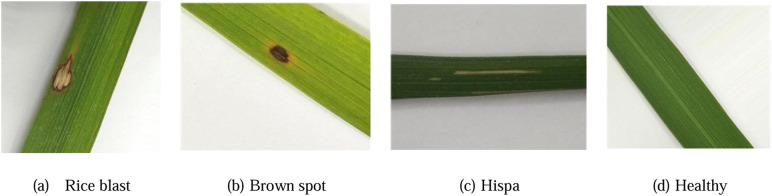
Samples of the dataset.

In this research, the rice leaf dataset was sourced from Do [27], which played a significant role in validating our proposed model. As shown in Fig 1, the database encompasses healthy leaves as well as three types of diseases, namely rice blast, brown spot, and hispa. From this dataset, 500 images of rice blast, 500 images of brown spot, 500 images of hispa, and 600 images of healthy leaves were gathered. Altogether, the total number of images amounted to 2100. These images were partitioned into the original training set and test set at a 9:1 ratio.

Data augmentation refers to the process of expanding the dataset in order to improve the performance of the model by creating various forms of images. Moreover, it is beneficial for alleviating overfitting during the model training process. Multiple data augmentation techniques were employed, including rotation transformations, horizontal and vertical flips, and intensity disturbance, among others. Through the application of these approaches, six new images were generated from each original image. Eventually, a dataset consisting of 14,700 images was successfully created with the help of the data augmentation technique. Specifically, the training set comprises 1890 × 7 = 13,230 images, while the test set contains 210 × 7 = 1,470 images.

**Fig 2:**
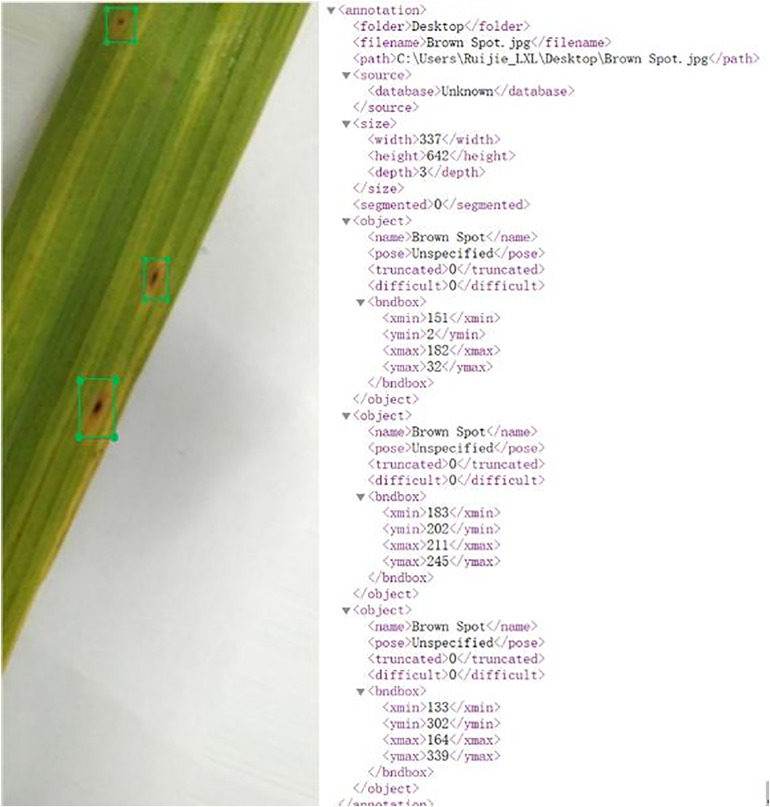
The image annotation result in XML file.

During the training process, 13,230 images were labeled for each of the following classes: healthy, rice blast, brown spots, and hispa. Meanwhile, the remaining images were utilized to evaluate the performance of our model. The distribution results are demonstrated in Table 1. For every image within the training dataset, a file was established based on Pascal VOC. The generated XML file encompassed details like the coordinate values of the bounding box and the types of diseases. Fig 2 shows an annotated image.

**Table 1:** Distribution of the dataset.

Leaf condition	Training set	Testing set
Rice blast	3150	350
Brown spot	3150	350
Hispa	3150	350
Healthy	3780	420

The experiment was carried out by leveraging the PyTorch deep learning framework under the following configuration: an Intel Core i5-11500 CPU, 128 GB of memory, along with an Nvidia RTX4080 GPU. Cuda 10.2 and Cudnn 7.51 were employed to expedite the GPU computation.

## 2 Methodology

**Fig 3:**
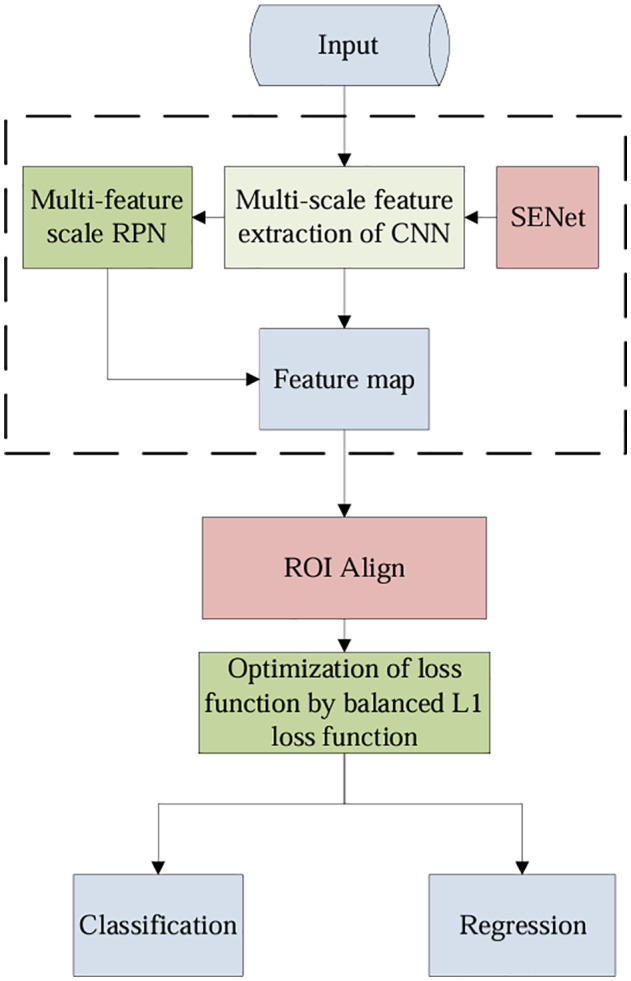
Flow of improved algorithm.

Faster-RCNN is composed of a feature extraction network, a region generation (RPN) module, and a target classification and position regression (RoiHead) module. The RPN module generates the target candidate region by sharing the feature layer and uses the full convolution network structure to classify and regress the quantitative region for each image. The RoiHead module performs target classification and location regression correction on the generated candidate regions on the high-level semantic feature map. However, there are still some shortcomings when using the original Faster R-CNN to detect rice leaf diseases. For example, RPN network uses the information obtained from convolution of the last layer feature map for subsequent operations, which may lead to insufficient utilization of small objects’ features and lower detection accuracy. Positioning errors are introduced through two quantization operations in the ROI-pooling layer. In addition, there are issues, such as poor detection performance owing to uneven data distribution. Based on this, the Faster R-CNN algorithm is optimized by selecting the ResNeXt50 network as the backbone and the ResNeXt module for feature extraction. Specifically, to improve the detection accuracy of dense and small objects such as brown spots, SENet attention mechanism modules are embedded into the backbone of Faster-RCNN for it to learn global features and automatically calibrate the weights of each channel, thereby enhancing key information, suppressing inconsequential information, and enhancing model detection performance with almost no increase in the parameter count. By multi-feature scale fusion, feature information of small objects can be mapped into the feature maps, and the feature maps contain richer semantic information, thereby improving detection accuracy. Additionally, ROI Align is used to eliminate quantization errors introduced during the pooling process. Moreover, a balanced L1 loss function is obtained by redesigning the smooth L1 loss function to reduce effectively the imbalance between difficult-to-learn samples with large gradients and easy-to-learn samples with small gradients. This allows for better training and improves detection performance. The structure of the improved Faster-RCNN (Faster-RCNN-Pro) is shown in Fig 3.

### 2.1 Improvement of the backbone with SENet embedded

Based on ResNet, ResNeXt draws inspiration from inception structures and adopts group convolutions, which can improve the network’s ability to extract features while reducing computational complexity. Therefore, to reduce the number of parameters while maintaining the detection effect, the ResNeXt50 network is selected as the backbone network and the ResNeXt module as the feature extraction module.

When original Faster R-CNN is used to detect lesions in fundus images, brown spots are often misdetected or unnoticed owing to an imbalance in distribution of confidence. So the weights of each channel must be calibrated in order to enable the network to learn global features and then improve detection accuracy of dense and small lesions. SENet [28] is a network that can emphasize information features selectively and suppress unnecessary features according to global information. By means of allowing the network to learn feature weight according to the loss and then assigns a weight value to each feature channel according to the importance, SENet can obtain the importance of each feature map. As a result, the network can focus on certain feature maps, which results in significant weight for effective feature maps and small weight for ineffective or ineffective feature maps, thus enabling the model to achieve better results. Therefore, to improve the accuracy of detecting dense small target objects, SENet attention mechanism modules are embedded into the backbone network allowing global features to be learned and automatically calibrate the weights of each channel, strengthen key information, and suppress inconsequential information; thus, the detection performance of the model is enhanced with almost no increase in the number of parameters.

**Fig 4:**
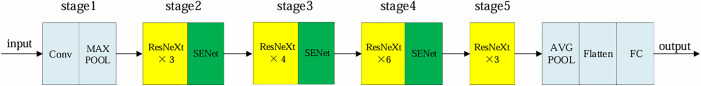
Structure of ResNeXt50 embedded with SENet.

ResNeXt50, which is adopted as the backbone network, consisted of five stages. Owing to the large number of feature map channels in Stage 5, two fully connected layers in the SENet attention mechanism module generate an excessive number of parameters, which reduces the detection speed. Therefore, the attention mechanism module is not embedded in Stage 5 of the backbone network. Specifically, the SENet attention mechanism modules are embedded before the shortcut connection of the last residual module in the second, third, and fourth stages of the backbone network ResNeXt50 (i.e., after the 10th, 22nd, and 40th layers of the backbone network). Consequently, key information of image is enhanced, background information is suppressed, and the confidence in objects that are difficult to identify is improved. The structure of ResNeXt50 embedded in SENet is shown in Fig 4.

The fundus image is processed in the backbone network of ResNeXt50 with embedded SENet attention mechanism modules for feature extraction and the output feature maps from stages 2–5 are selected for the subsequent calculations. Consequently, the initial feature maps are obtained in four scales including {C2: 256 × 256, C3: 128 × 128, C4: 64 × 64, and C5: 32 × 32}. Faster-RCNN optimized using this strategy is termed Faster-RCNN-I.

### 2.2 Multi-feature scale fusion

RPN network uses the information obtained by the convolution of the last layer of the feature map as the feature map for subsequent operations, which prevents the features of small targets from being fully utilized and is apparently significantly unfavorable to the automatic detection of brown spots. Therefore, the multi-feature scale fusion method is adopted to improve the feature extraction network and RPN structure; the Faster-RCNN of this improved structure is called Faster-RCNN-II.

Multi-feature scale fusion integrates the spatial feature dimensions of a deep network feature map with high-level semantic information in a certain manner so it is the same as that of a shallow network feature map with low-level semantic information, and then merges these in an additive manner, thereby enabling the extracted feature map to have more abundant semantic information for subsequent use [29]. Following multi-feature scale fusion, it needs to be improved in three layers including the network structure, detection and classification layer, and interest pooling layer.

**Fig 5:**
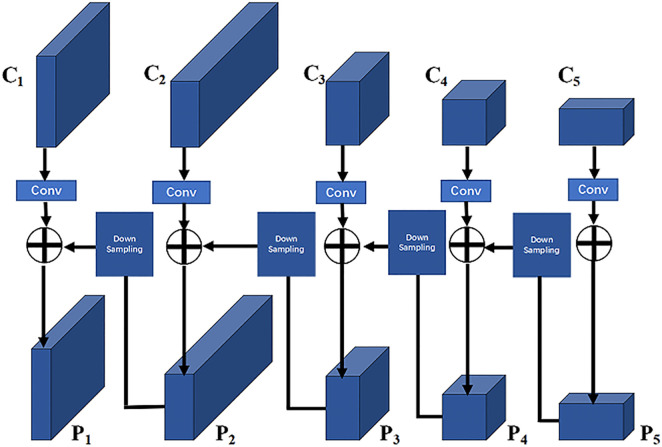
Multi-feature scale fusion.

For the feature extraction backbone network, each down-sampling of the convolution layer is called a stage. As shown in Fig 5, the backbone network can be divided into five stages; namely C1, C2, C3, C4, and C5. For $C_{\text{i}}\;\left(\text{i}=\text{1,2,3,4,5}\right)$, it matches and adds with the scale of Ci−1 by the 1 × 1 convolution operation and down-sampling, and finally eliminates the aliasing effect caused by the multi-dimensional feature discontinuity following addition by the 3 × 3 convolution operation, thereby obtaining a new feature map $P_{i}\;\left(\text{i}=\text{1,2,3,4,5}\right)$.

**Fig 6:**
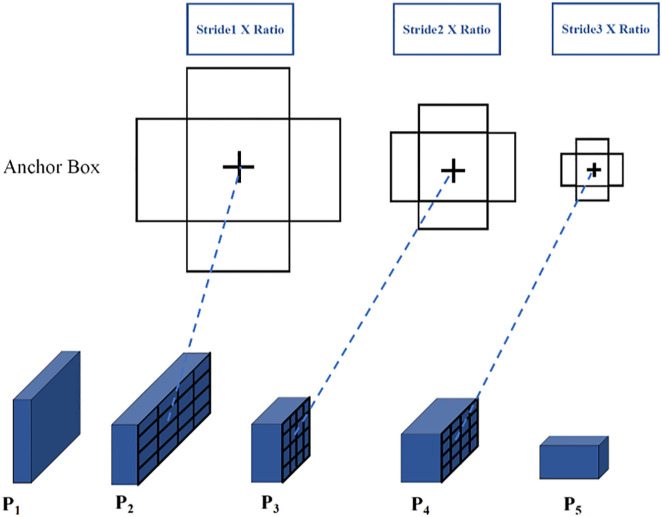
RPN with multi-feature fusion.

The RPN network calculation adopts the feature map of the last layer of the network and classifies and regresses the *k* anchor boxes mapped from the original map to the feature map. These *k* anchor boxes are preset to a fixed aspect ratio and are calculated in RPN using the feature map of the same stage. To fuse multiple feature scales, the anchor box matches the corresponding anchor box on different feature maps while maintaining the original preset size and scale. Considering the calculation efficiency, the anchor boxes are distributed on the three feature layers of P2, P3, and P4 respectively. The anchor boxes allocated on each feature map have the following three scales: 1:1, 1:2 and 2:1. In RPN detection, the anchor box of this layer is used if the target can be classified on this feature map. The specific process is shown in Fig 6.

After changing the region suggestions generated by RPN, the pooling mode of the feature map needs to be adjusted accordingly. ROI pooling in the original faster- RCNN considers the last layer of the feature map as the input, where the mapping relationship between the feature map and RPN is as follows:


$$P=4+{log}_{\text{2}}\frac{\sqrt{wh}}{k}$$ (1)

Here, *P* represents the level of the feature map; a larger *P* value indicates a smaller scale of the corresponding feature map. The number 4 represents the input level, *k* is the input image scale, and *w* and *h* denote the width and length of the ROI region, respectively. Formula (1) demonstrates that the input of ROI pooling is single. By using the ROI region extracted by RPN on the last layer of the feature map as the input, the ROI region obtained by the multi-feature scale RPN cannot be effectively used. Therefore, ROI pooling needs to be improved, as shown in formula (2):


$$\left\{ \begin{array}{c} @l@P=P_{i}+{log}_{\text{2}}\frac{\sqrt{wh}}{k}\\ P_{i}=\left\{P_{i+1},P_{i}\right\} \end{array}\right.$$ (2)

Here, the input feature map is the result obtained by summing the feature map of the current layer and the small-scale feature map of the deep layer through down-sampling. Using the feature map corresponding to the ROI region obtained by RPN on multiple feature scales as the input of ROI pooling ensures that the target features can be mapped onto the feature map, thus improving the target detection accuracy.

### 2.3 ROI align

In the original Faster-RCNN, ROI Pooling is used to pool candidate regions of different sizes into feature maps of the same size, which generates two quantization errors and will cause the candidate regions to significantly deviate from the position on the original regression feature map. This deviation has a critical impact on the detection of small targets and is referred to as the misalignment effect [30].

ROI Align is a regional feature aggregation method proposed in the mask RCNN framework that can solve the misalignment problem caused by the two quantization errors in ROI pooling [30]. ROI Align uses bilinear interpolation to obtain the image values on the pixel points whose coordinates are floating-point numbers, thus transforming the entire feature aggregation process into a continuous operation. ROI Align does not require quantization and retains floating-point number operations, which enables the entire network to obtain a better detection accuracy. The improvement of Faster-RCNN by ROI Align can effectively improve the detection performance of the network for small objects. Faster-RCNN optimized using this strategy is termed Faster-RCNN-III.

### 2.4 Optimization of loss function

The loss function of Faster-RCNN includes classification and regression losses, as shown in formula (3) [31]:


$$\text{L}(\{p_{i}\},\{t_{i}\})=\frac{\text{1}}{N_{cls}}\sum_{i}L_{cls}\left(p_{i},\overset{*}{\underset{i}{p}}\right)+\lambda \frac{\text{1}}{N_{reg}}\sum_{i}\overset{*}{\underset{i}{p}}L_{reg}\left(t_{i},\overset{*}{\underset{i}{t}}\right)$$ (3)

where subscript *i* indicates the number of the candidate region, which changes with the number of true values in the same batch. *P*_*i*_ is the discrete probability distribution in the detection network. P*_i_ is the GT calibration value of the *ith* frame, that is, the true value. $\overset{*}{\underset{i}{p}}=\text{1}$ when the target object exists in the frame; conversely, $\overset{*}{\underset{i}{p}}=\text{0}$. The classification loss $L_{cls}$ and regression loss $L_{reg}$ are normalized by $N_{cls}$, $N_{\text{reg}}$, and weight λ, respectively. $t_{i}=\left\{t_{x},t_{y},t_{w},t_{h}\right\}$ indicates the predicted value of the target object position and $\overset{*}{\underset{i}{t}}=\left\{\overset{*}{\underset{x}{t}},\overset{*}{\underset{y}{t}},\overset{*}{\underset{w}{t}},\overset{*}{\underset{h}{t}}\right\}$ is the supervision information manually provided in the learning sample.

Considering the regression loss, because the parameter regression is approximately linear and the noise in the annotation information is relatively high, the gradient return is unstable [31]. To alleviate this situation, a smooth L1 loss function with a gentle gradient is used for the regression loss, as shown in equations (4) and (5):


$$L_{\text{reg}}\left(t_{i},\overset{*}{\underset{i}{t}}\right)=R(t_{i}-\overset{*}{\underset{i}{t}})$$ (4)

$$smooth_{L_{\text{1}}}\left(x\right)=\left\{\begin{array}{c} @l@0.5x^{\text{2}}\times 1/\sigma^{\text{2}},\;\left|x\right|<1/\sigma^{\text{2}}\\ \left|x\right|-0.5\;,\text{ otherwise} \end{array}\right\}$$ (5)

where *R* in formula (4) is the smooth L1 loss function in formula (5). The parameter σ is used to control the range of the smoothing region to avoid the excessive influence of the large gradient on the network parameters.

The gradient of equation (5) is calculated as follows:


$$\frac{\partial L_{smoothL_{\text{1}}}}{\partial x}=\left\{ \begin{array}{c} @l@x,\left|x\right|\leq 1\\ 1,otherwise \end{array}\right.$$ (6)

In back propagation, loss is defined by the difficult samples; namely, the loss of easy samples is small. Therefore, the smooth L1 function in equation (6) demonstrates that the difficult samples provide more gradient information in comparison to the easy samples, which leads to the imbalance in the learning ability between the difficult and easy samples.

The balanced L1 loss function is designed to solve this problem, that is, the gradient function of the balanced L1 loss is obtained from equation (6), as shown in equation (7):


$$\begin{array}{c} \frac{\partial L_{{\text{BalancedL}}_{\text{1}}}}{\partial x}=\left\{ \begin{array}{c} @c@0.3ln\left(b\left|x\right|+1\right),\begin{array}{cc} & \left|x\right|\leq 1 \end{array}\\ \gamma ,\begin{array}{cc} & otherwise \end{array} \end{array}\right. \end{array}$$ (7)

Here, γ is considered as the gradient for the samples with a gradient higher than γ during back propagation to balance the gradient values of the samples with a small gradient and those that are difficult to learn. Based on the gradient information in equation (7), the equilibrium L1 loss function is obtained by integration as follows:


$$L_{\text{BalancedL1}}\left(x\right)=\left\{ \begin{array}{c} @l@\frac{0.3c}{b}(b\left|x\right|+1)ln(b\left|x\right|+1)-0.3\left|x\right|,\left|x\right|<1\\ \gamma \left|x\right|+d\text{, otherwise} \end{array}\right.$$ (8)

To satisfy the subsection function and provide a gradient at the subsection in equation (8), $b=exp\left(\frac{\gamma }{0.3}\right)$. On the Kaggle DR dataset, γ is 1.

The balanced L1 loss function can effectively reduce the imbalance between the samples that have a large gradient and are difficult to learn, and those with a small gradient that are easy to learn, thus obtaining a model that is better trained. The Faster-RCNN that is optimized by this strategy is called Faster-RCNN-IV.

## 3 Results and discussion

### 3.1 System performance evaluation

The precision rate (*P*), recall rate (*R*), mean average precision (mAP) values, and F1-score were used to evaluate the model and verify the practical application of the proposed Faster-RCNN-Pro model.

### 3.2 Analysis of experimental results

#### 3.2.1 Training parameters.

During the training process of the proposed Faster-RCNN-Pro network model with the constructed dataset, the batch processing size is configured as 2. A total of 31,000 iterations are carried out, with the initial basic learning rate set at 0.00125. After 23,000 iterations, the learning rate decays to 0.000125. The optimization approach adopts the mini-batch stochastic gradient descent method with the momentum factor. Meanwhile, for the feature extraction network, the model on the COCO dataset is utilized as the pre-training model.

The loss function of the Faster-RCNN-Pro exhibits a convergence tendency within 20 training epochs. At the start of the training process, the loss function drops rapidly. After 22,000 iterations, it begins to converge slowly while experiencing fluctuations. Once the number of iterations reaches 20,000, the learning rate is reduced by a factor of 10, and both the loss function value and its fluctuations decrease as well. The loss function converges smoothly until 30,000 iterations, and the recognition performance is satisfactory. Hence, the model obtained after the 31,000th iteration is regarded as the final detection model.

#### 3.2.2 Analysis of module effectiveness.

To analyze the impact of the proposed 4-point improvement strategy on the detection performance of Faster RCNN, ablation experiments are conducted to compare and analyze its automatic detection performance with various improved structures on the constructed dataset for rice leaf diseases. The specific detection performance is shown in Table 2. As Table 2 shows, each improvement strategy increases the detection performance of original Faster RCNN to varying degrees. Specifically, key information in an image can be enhanced, background information can be suppressed, and the confidence of objects that are difficult to recognize can be improved by embedding attention mechanism modules into the backbone network for feature extraction. Specifically, Faster RCNN-I’s mAP increased by 6.96% compared to the baseline, Faster RCNN. By multi-feature scale fusion, feature information of rice leaf diseases can be mapped into the feature maps, and the feature maps contain richer semantic information, thereby improving detection accuracy of them. Specifically, the mAP of Faster RCNN-II increased by 8.01% compared to that of the baseline Faster RCNN. The model’s performance is improved by ROI Align which provided more accurate information on features from the prior bounding boxes. Specifically, the mAP of Faster RCNN-III increased by 7.58% compared to the baseline. The optimized loss function is used to balance the gradient in the model, which is provided by samples that are difficult and easy to learn and reduce the impact of noise. Therefore, the mAP of Faster RCNN-IIII is 6.04% higher than that of the baseline. In particular, the detection performance of Faster-RCNN-Pro, which is adopted by all four improvement strategies, is more noticeably improved, and its mAP is increased by 14.90% compared to that of the baseline.

**Table 2:** Performance evaluation of various improved Faster-RCNN models on the test set.

Network structure	SENet	Multi-feature scale fusion	ROI Align	Balanced L1 loss function	P /%	R /%	mAP@0.5/%
Faster-RCNN	×	×	×	×	80.52	72.16	74.31
Faster-RCNN-I	√	×	×	×	83.93	75.65	81.27
Faster-RCNN-II	×	√	×	×	87.05	79.17	82.32
Faster-RCNN-III	×	×	√	×	82.17	78.02	81.89
Faster-RCNN-IV	×	×	×	√	81.95	79.91	80.35
**Faster-RCNN-Pro**	√	√	√	√	92.21	91.30	89.21

#### 3.2.3 Comparative analysis of different algorithms.

To confirm the effectiveness and robustness of the proposed method, we compared it with state-of-the-art methods in terms of Precision, Recall, F1-score, and mAP. Training and testing were conducted for 1000 iterations using the same dataset and parameter settings. To further demonstrate the superiority of our method, we present the quantitative detection results for the three rice leaf diseases in Table 3. The compared methods include SVM [5], CNN [20], Faster-RCNN [25], and YOLO V5+ [26]. Faster-RCNN-Pro was superior to the other methods in accuracy of detecting the three types of rice leaf diseases. Although YOLO V5 + achieved state-of-the-art weedy rice detection results, it failed to transfer well to rice leaf diseases. Specifically, as illustrated in Table 3, the proposed method outperforms YOLO V5+ in mAP@0.5 by approximately 1%. Notably, however, a significant discrepancy emerges between the two methods in terms of the *R*—a critical indicator for disease detection. The proposed method achieves the *R* of 91.30%, compared to 87.86% for YOLO V5 + , representing an improvement of 3.44%. For rice leaf disease detection, *R* is directly tied to the effectiveness of field-based disease prevention and control. A low *R*, indicative of excessive missed detections, can lead to the spread of undetected lesions, thereby causing substantial crop losses. This *R*-centric performance advantage holds far greater practical significance for real-world field disease management than the 1% difference in mAP, as it more effectively mitigates economic losses arising from missed inspections.

**Table 3:** Detection result comparison of the proposed model against state-of-the-art models.

Methods	P/%	R/%	F1-score/%	mAP@0.5/%
SVM [5]	84.50	79.22	81.77	–
CNN [20]	89.13	82.45	85.66	81.73
Faster-RCNN [25]	91.91	86.62	89.19	84.92
YOLO V5+ [26]	92.50	87.86	90.12	88.15
**Proposed Faster-RCNN-Pro**	92.21	91.30	91.75	89.21

In addition, the proposed Faster-RCNN-Pro is tailored to the unique requirements of rice leaf disease detection, with a particular focus on enhancing disease classification accuracy. Specifically, by integrating the SENet attention mechanism, the model adaptively recalibrates channel-wise weights, strengthens the discriminative features of distinct diseases, and mitigates inter-class confusion. As presented in Table 3, the F1-score of the proposed Faster-RCNN-Pro reaches 91.75%, outperforming YOLOv5+ (90.12%). This result directly demonstrates the model’s superior capability in fine-grained disease category discrimination. For practical agricultural applications, the accurate identification of disease types is a critical prerequisite for farmers to select targeted pesticides and implement effective disease control measures.

Therefore, the proposed rice leaf disease identification method based on Faster-RCNN-Pro exhibits notable advantages in mitigating missed detection risks and facilitating precise, targeted disease control. These merits enable effective addressing of the core challenges of “missed detection, misclassification, and improper pesticide application” encountered by farmers in practical disease prevention and management.

**Fig 7:**
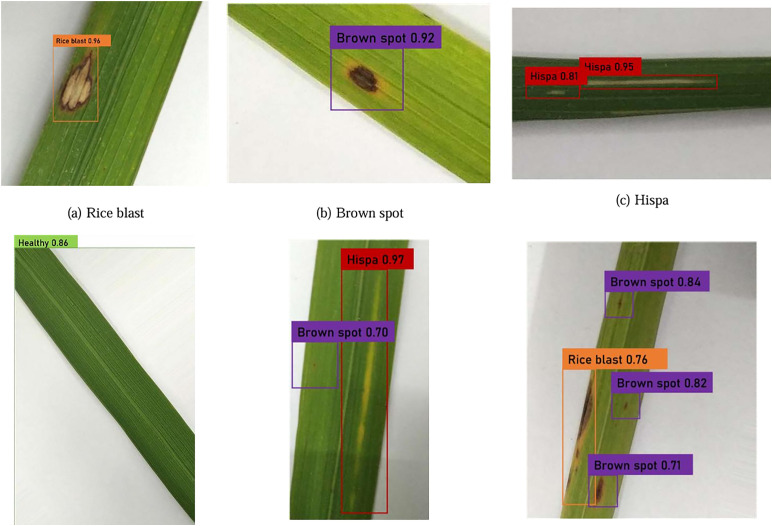
Detection results.

The results of the identification of rice leaf diseases are shown in Fig 7. The proposed approach can identify both a single object and multiple objects of a single class as well as multiple objects of multiple classes. Therefore, the proposed method demonstrates high detection performance in both single- and multi-class assessments. Especially in recognizing the small features of lesions, it can handle rice leaf disease detection tasks with numerous small targets effectively, as shown in Fig 7(f).

## 4 Conclusions

Currently, there is significant room for improvement in both the speed and accuracy of rice leaf disease detection. The proposed Faster-RCNN-Pro algorithm offers a new approach to improve the accuracy of rice leaf disease recognition. Faster-RCNN-Pro makes the following main contributions. Based on Faster-RCNN, the algorithm enhances the confidence of objects that are difficult to recognize by embedding SENet attention modules in the backbone of Faster-RCNN, it adopts multi-feature scale fusion to improve the utilization of features for small targets, ROI Align is used to reduce quantization errors, and the loss function is optimized to effectively reduce the imbalance between the samples that have a large gradient and are difficult to learn, and those with a small gradient that are easy to learn, to achieve the optimization of Faster-RCNN for the accurate detection of rice leaf diseases. This algorithm not only meets the requirements for real-time and robust detection but also exhibits excellent detection accuracy. Therefore, this algorithm can effectively identify rice leaf diseases. Based on this, farmers can develop science-based pesticide application regimens in accordance with the high-precision diagnostic outcomes provided. When multiple diseases are induced by the same pathogen, broad-spectrum targeted pesticides may be selected to avoid elevated costs and phytotoxicity risks associated with mixing multiple pesticide formulations. In cases where multiple diseases stem from different pathogens, scientific tank-mixing or staged application strategies should be implemented. The model-guided precision pesticide application regimens enable targeted prevention and control, which reduces ineffective pesticide application, mitigates phytotoxicity risks, and curbs the impairment of leaf photosynthetic function caused by multiple diseases. In comparison with conventional pesticide application practices, this approach can significantly boost rice yields. Consequently, this research not only cuts pesticide procurement costs and alleviates environmental pollution arising from excessive pesticide use but also improves rice productivity, which is highly aligned with the development demands of green agriculture in developing countries.

The Faster-RCNN-Pro that has been put forward has achieved remarkable progress when it comes to detection accuracy. However, its real-time detection performance needs to be improved. The reason for this lies in the fact that the network model has a large number of parameters and high computational complexity. Consequently, it is worthwhile to explore approaches to further simplify the network model. This is of vital importance for the establishment of a dynamic and automatic system which is based on mobile terminal processors and components of the agricultural Internet of Things, aiming to recognize large-scale rice leaf diseases. Moreover, it is beneficial for the modernization of the agricultural industry.
